# Effectiveness of BNT162b2 and CoronaVac vaccinations against SARS-CoV-2 omicron infection in people aged 60 years or above: a case–control study

**DOI:** 10.1093/jtm/taac119

**Published:** 2022-10-17

**Authors:** Eric Yuk Fai Wan, Anna Hoi Ying Mok, Vincent Ka Chun Yan, Cheyenne I Ying Chan, Boyuan Wang, Francisco Tsz Tsun Lai, Celine Sze Ling Chui, Xue Li, Carlos King Ho Wong, Chak Sing Lau, Ian Chi Kei Wong, Esther Wai Yin Chan

**Affiliations:** Department of Pharmacology and Pharmacy, Li Ka Shing Faculty of Medicine, Centre for Safe Medication Practice and Research, The University of Hong Kong, Hong Kong Special Administrative Region, China; Laboratory of Data Discovery for Health (D24H), Hong Kong Science and Technology Park, Hong Kong Special Administrative Region, China; Department of Family Medicine and Primary Care, School of Clinical Medicine, Li Ka Shing Faculty of Medicine, The University of Hong Kong, Hong Kong Special Administrative Region, China; Department of Family Medicine and Primary Care, School of Clinical Medicine, Li Ka Shing Faculty of Medicine, The University of Hong Kong, Hong Kong Special Administrative Region, China; Department of Pharmacology and Pharmacy, Li Ka Shing Faculty of Medicine, Centre for Safe Medication Practice and Research, The University of Hong Kong, Hong Kong Special Administrative Region, China; Department of Family Medicine and Primary Care, School of Clinical Medicine, Li Ka Shing Faculty of Medicine, The University of Hong Kong, Hong Kong Special Administrative Region, China; Department of Family Medicine and Primary Care, School of Clinical Medicine, Li Ka Shing Faculty of Medicine, The University of Hong Kong, Hong Kong Special Administrative Region, China; Department of Pharmacology and Pharmacy, Li Ka Shing Faculty of Medicine, Centre for Safe Medication Practice and Research, The University of Hong Kong, Hong Kong Special Administrative Region, China; Laboratory of Data Discovery for Health (D24H), Hong Kong Science and Technology Park, Hong Kong Special Administrative Region, China; Laboratory of Data Discovery for Health (D24H), Hong Kong Science and Technology Park, Hong Kong Special Administrative Region, China; School of Nursing, Li Ka Shing Faculty of Medicine, The University of Hong Kong, Hong Kong Special Administrative Region, China; School of Public Health, Li Ka Shing Faculty of Medicine, The University of Hong Kong, Hong Kong Special Administrative Region, China; Department of Pharmacology and Pharmacy, Li Ka Shing Faculty of Medicine, Centre for Safe Medication Practice and Research, The University of Hong Kong, Hong Kong Special Administrative Region, China; Laboratory of Data Discovery for Health (D24H), Hong Kong Science and Technology Park, Hong Kong Special Administrative Region, China; Department of Medicine, School of Clinical Medicine, Li Ka Shing Faculty of Medicine, The University of Hong Kong, Hong Kong Special Administrative Region, China; Department of Pharmacology and Pharmacy, Li Ka Shing Faculty of Medicine, Centre for Safe Medication Practice and Research, The University of Hong Kong, Hong Kong Special Administrative Region, China; Laboratory of Data Discovery for Health (D24H), Hong Kong Science and Technology Park, Hong Kong Special Administrative Region, China; Department of Family Medicine and Primary Care, School of Clinical Medicine, Li Ka Shing Faculty of Medicine, The University of Hong Kong, Hong Kong Special Administrative Region, China; Department of Medicine, School of Clinical Medicine, Li Ka Shing Faculty of Medicine, The University of Hong Kong, Hong Kong Special Administrative Region, China; Department of Pharmacology and Pharmacy, Li Ka Shing Faculty of Medicine, Centre for Safe Medication Practice and Research, The University of Hong Kong, Hong Kong Special Administrative Region, China; Laboratory of Data Discovery for Health (D24H), Hong Kong Science and Technology Park, Hong Kong Special Administrative Region, China; Research Department of Practice and Policy, School of Pharmacy, University College London, London, UK; Aston Pharmacy School, Aston University, Birmingham, UK; Department of Pharmacy, The University of Hong Kong-Shenzhen Hospital, Shenzhen, China; Department of Pharmacology and Pharmacy, Li Ka Shing Faculty of Medicine, Centre for Safe Medication Practice and Research, The University of Hong Kong, Hong Kong Special Administrative Region, China; Laboratory of Data Discovery for Health (D24H), Hong Kong Science and Technology Park, Hong Kong Special Administrative Region, China; Department of Pharmacy, The University of Hong Kong-Shenzhen Hospital, Shenzhen, China; The University of Hong Kong Shenzhen Institute of Research and Innovation, Shenzhen, China

**Keywords:** COVID-19, older adults, vaccine effectiveness, Omicron BA.2, Comirnaty, Sinovac, elderly

## Abstract

**Background:**

In view of limited evidence that specifically addresses vaccine effectiveness (VE) in the older population, this study aims to evaluate the real-world effectiveness of BNT162b2 and CoronaVac in older adults during the Omicron BA.2 outbreak.

**Methods:**

This case–control study analyzed data available between January and March 2022 from the electronic health databases in Hong Kong and enrolled individuals aged 60 or above. Each case was matched with up to 10 controls by age, sex, index date and Charlson Comorbidity Index for the four outcomes (COVID-19 infection, COVID-19-related hospitalization, severe complications, and all-cause mortality) independently. Conditional logistic regression was conducted to evaluate VE of BNT162b2 and CoronaVac against COVID-19-related outcomes within 28 days after COVID-19 infection among participants stratified by age groups (60–79, ≥80 years old).

**Results:**

A dose–response relationship between the number of vaccine doses received and protection against severe or fatal disease was observed. Highest VE (95% CI) against COVID-19 infection was observed in individuals aged ≥80 who received three doses of BNT162b2 [75.5% (73.1–77.7%)] or three doses of CoronaVac [53.9% (51.0–56.5%)] compared to those in the younger age group who received three doses of BNT162b2 [51.1% (49.9–52.4%)] or three doses of CoronaVac [2.0% (−0.1–4.1%)]. VE (95% CI) was higher for other outcomes, reaching 91.9% (89.4–93.8%) and 86.7% (84.3–88.8%) against COVID-19-related hospitalization; 85.8% (61.2–94.8%) and 89.8% (72.4–96.3%) against COVID-19-related severe complications; and 96.4% (92.9–98.2%) and 95.0% (92.1–96.8%) against COVID-19-related mortality after three doses of BNT162b2 and CoronaVac in older vaccine recipients, respectively. A similar dose–response relationship was established in younger vaccine recipients and after stratification by sex and Charlson Comorbidity Index.

**Conclusion:**

Both BNT162b2 and CoronaVac vaccination were effective in protecting older adults against COVID-19 infection and COVID-19-related severe outcomes amidst the Omicron BA.2 pandemic, and VE increased further with the third dose.

## Introduction

The severe acute respiratory syndrome coronavirus 2 (SARS-CoV-2) has been circulating globally with its Omicron subvariants, which caused a surge in cases at the beginning of the year and has been increasingly difficult to contain as compared to previous variants.^1^^,^^2^ According to the World Health Organization, Omicron BA.2 was the dominant variant circulating globally in the recent outbreak, accounting for more than 86% of all sequenced COVID-19 cases in early 2022, and has been classified as a variant of concern.^3^ Research suggested that some of Omicron’s enhanced transmissibility could come from its evasion of neutralizing antibodies, especially in people who were previously infected, but not vaccinated.^4^ Thus, it is of public health importance to assess how well existing vaccines protect against Omicron BA.2.

Although the safety and efficacy of COVID-19 vaccines in older people is critical, most clinical trials excluded this group, especially those with comorbidities and frailty, leaving insufficient published data on safety and efficacy in this population.^5^ The older population is of greater concern for their higher risk of severe outcomes and mortality with age,^6–8^ weaker vaccine-induced immune responses in comparison with younger individuals,^9–11^ as well as lower neutralization potency against the wild-type virus and the variants of concern.^9^^,^^12^ While the older population has been affected the most and experienced the worst outcomes, there is lacking research on vaccination effectiveness against Omicron subvariant for this specific age group. Of all deaths recorded in the recent wave dominated by Omicron BA.2 in Hong Kong, the high overall mortality rate was mainly driven by deaths among unvaccinated or vaccinated persons aged ≥60 years.^13^ Studies concluded high effectiveness from two doses of mRNA vaccines and substantial additional protection from a third dose against Omicron in older adults.^14^^,^^15^ Evidence on the effectiveness of boosting with heterologous vaccines against the more transmissible Omicron BA.2 remains insufficient, as are data on the performance of inactivated vaccines, and in particularly the highly vulnerable population.

Currently, two COVID-19 vaccines, including Fosun-BioNTech’s BNT162b2 (Pfizer-BioNTech, mRNA vaccine) and Sinovac Biotech (HK) Limited CoronaVac (inactivated vaccine), have been authorized for emergency use in Hong Kong. Previous large-scale randomized trials and observational studies showed that a primary series of BNT162b2 or CoronaVac conferred high protection against COVID-19 and related mortality.^16–19^ Some observational evidence suggests strong and durable protection against severe disease and death from both mRNA and inactivated vaccines, with transient protection against milder symptomatic disease.^20^^,^^21^ However, most existing vaccine-effectiveness estimates focused primarily on mRNA vaccine as the exposure, whilst inactivated vaccines have also been approved for emergency use.^22^ A recent study demonstrated the effectiveness of both BNT162b2 mRNA and inactivated CoronaVac vaccines against Omicron BA.2, yet falls short of demonstrating to demonstrate the vaccine effectiveness of heterologous booster vaccination.^23^ Moreover, Hong Kong records a low vaccine uptake in people aged ≥60 years, with only 21.5% (15.3% with CoronaVac and 6.2% with BNT162b2) and 27.9% (12.2% with CoronaVac and 15.7% with BNT162b2) of people who have received two and three doses of vaccine, respectively, as at 31 March 2022.^24^ Given the general low-vaccine coverage among older adults as observed in other regions, such as Bangladesh, Canada, and Australia,^25–28^ generalizability of the findings utilizing data gathered from general population remains a question.

Globally, rates of COVID-19 infection and severe complications after infection remain high.^29^ In Hong Kong, albeit the implementation of multi-pronged containment measures including test-and-trace, isolation of confirmed cases, quarantine of close contacts and inbound travellers in addition to border control, community-wide social distancing measures and mask mandate in public places, the arrival of the Omicron BA.2 exhibited high superspreading potential with sustained transmissibility due to its growth advantage over other variants,^30^^,^^31^ thus triggering the fifth wave of outbreak in Hong Kong. As a result, the cumulative incidence and mortality rate shot up in Hong Kong and the city ended up being in an unenviable position of having the highest mortality rate in the developed world.^32^^,^^33^ This posts questions to the effectiveness of the available vaccines in protecting older adults. In view of that, this study aims to investigate the effectiveness of both vaccines against Omicron BA.2 infection as well as its severity and mortality at the population level in Hong Kong using real-world data, particularly in older adults.

## Methods

### Study design and population

Individuals aged 60 and over who did not have a history of COVID-19 infection were included in this case–control study. Patients who received their last dose of vaccine more than 180 days before the index date were excluded from the analysis since waning immunity after vaccination is well-recognized after six months, as described in previous studies.^34^^,^^35^ Data on patient demographics were obtained from the electronic health database of the Hong Kong Hospital Authority (HA), and records on previous COVID-19 infection and vaccination were obtained from the Department of Health (DH) of the Government of the Hong Kong Special Administrative Region. Obtained datasets were linked by unique personal identification numbers. These databases have previously been applied in several COVID-19 pharmacovigilance studies.^36–46^

### Definitions of vaccine exposure

The aforementioned Hong Kong COVID-19 vaccination programme was first implemented on 23 February 2021 to provide BNT162b2 and CoronaVac to individuals aged 16 years old or above free of charge. Booster shots were available to older people aged 60 or above since 11 November 2021.^47^ Further details on the rollout schedule are listed in Supplementary Table 1 available as Supplementary data at *JTM* online. Members of the public are advised to complete the first two doses of the series with the same product unless proof of exemption is provided, and are allowed to choose any of the covid-19 vaccines for boosting. This gives rise to homologous or heterologous COVID-19 vaccinations. Therefore, we categorized COVID-19 vaccinations into 9 groups according to the types of vaccine and the number of doses administered as follows: (i) unvaccinated, (ii) one dose of BNT162b2 only, (iii) one dose of CoronaVac only, (iv) two doses of BNT162b2 only, (v) two doses of CoronaVac only, (vi) three doses of BNT162b2, (vii) three doses of CoronaVac, (viii) two doses of BNT162b2 followed by a CoronaVac booster and (ix) two doses of CoronaVac followed by a BNT162b2 booster.

### Definitions of COVID-19 infection and complications

The outcomes of this study include (i) COVID-19 infection, as determined by polymerase chain reaction (PCR) test results; (ii) COVID-19-related hospitalization, defined as hospital admission within 28 days of COVID-19 infection; (iii) COVID-19-related severe complications, defined as admission to the intensive care unit (ICU) or use of ventilatory support within 28 days of infection, including intubation, mechanical ventilation, and oxygen supplementation, identified using the International Classification of Diseases, Ninth Revision, clinical modification (ICD-9-CM) procedure codes (39.65, 89.18, 93.90, 93.95, 93.96, 96.04, 96.7*x*) and (iv) COVID-19-related mortality, defined as all-cause mortality within 28 days after COVID-19 infection. Records on mortality were provided by the Hong Kong Deaths Registry, which officially records all registered deaths in Hong Kong.

### Matching method

We deliberately overlapped the period of inclusion (1 January 2022 to 31 March 2022) with that of the Omicron BA.2 outbreak in Hong Kong in order to assess the effectiveness of BNT162b2 and CoronaVac against Omicron BA.2.^33^ One case was matched up to 10 controls according to age category (five-year age bands), sex, index date (date of documented infection for case, and the date of hospitalization or attendance at outpatient clinic for control, ±3 calendar days) and Charlson Comorbidity Index (0, 1–2, 3–4, ≥5).^48^ The matching procedure was conducted independently for each outcome. For the outcome of (i) COVID-19 infection, subjects in the control group were those who attended HA services during the inclusion period who had neither a positive PCR test nor a positive rapid antigen test (RAT) result. This study defined the outcome of COVID-19 infection by PCR results only, since not all patients tested positive with RAT by themselves underwent a confirmatory PCR test. As of 31 March 2022, it was estimated that 11.7% of people aged 60–79 tested positive with PCR, and 11.2% of people aged ≥80 tested positive with PCR. Meanwhile, the estimated proportion of people who tested positive with RAT only was 4.8% in those aged 60–79 and 5.0% if those aged ≥80. For other COVID-19-related outcomes (i.e. (ii) COVID-19-related hospitalization, (iii) COVID-19-related severe complications, and (iv) COVID-19-related mortality), subjects in the control group were those who attended HA services during the inclusion period but did not experience the corresponding COVID-19-related outcome.

### Statistical analysis

Conditional logistic regression was used to estimate the vaccine effectiveness for each outcome among older vaccine recipients stratified by age groups (60–79, ≥80 years old). Chronic comorbidities (hypertension, cancer, diabetes mellitus, chronic kidney disease, respiratory disease, coronary heart disease, stroke, heart failure or dementia) and the use of chronic medications (renin-angiotensin-system agents, beta blockers, calcium channel blockers, diuretics, nitrates, lipid lowering agents, oral anticoagulants, antiplatelets, immunosuppressants, anti-diabetic drugs, insulin) were included as covariates in the model. Vaccine effectiveness (VE) was calculated using (1 − adjusted odds ratio [OR]) × 100%, where the adjusted OR was obtained in the conditional logistic regressions. The reference group for vaccination status was those who had not received their first vaccine dose before the date of sample collection.

Three sensitivity analyses were conducted in this study. The first sensitivity analysis excluded patients who developed infection <14 days after each dose of vaccine, as the time needed for vaccine effect to become fully in place is generally regarded as 14 days.^49–51^ The second sensitivity analysis included patients who received their last dose of vaccine more than 180 days before the index date since a few studies demonstrated durable antibody levels^52^ and a relatively long duration of protection against COVID-19-related mortality after vaccination.^53^^,^^54^ The third sensitivity analysis defined a positive COVID-19 case by either a positive PCR test or self-reported positive RAT result, in contrast to using PCR alone to define cases in the main analysis. Subgroup analyses stratified by sex (male; female) and Charlson Comorbidity Index (<4, ≥4) were conducted.

All statistical tests were two-sided, and *P* values <0.05 were considered statistically significant. Statistical analysis was conducted using R version 4.0.3 (www.R-project.org). At least two investigators (VKCY, BW, CIYC) conducted the statistical analyses independently for quality assurance. Strengthening the Reporting of Observational Studies in Epidemiology (STROBE) statement checklists were followed to guide transparent reporting of the case–control study.^55^

### Ethical approval

This study was approved by the Central Institutional Review Board of the Hospital Authority of Hong Kong (CIRB-2021-005-4) and the DH Ethics Committee (LM171/2021).

### Role of the funding source

The funder has no role in the study design, data collection, data analysis, data interpretation and writing of the report. The corresponding authors had full access to all the data in the study and took final responsibility for the decision to submit for publication.

## Results

**Figure 1:**
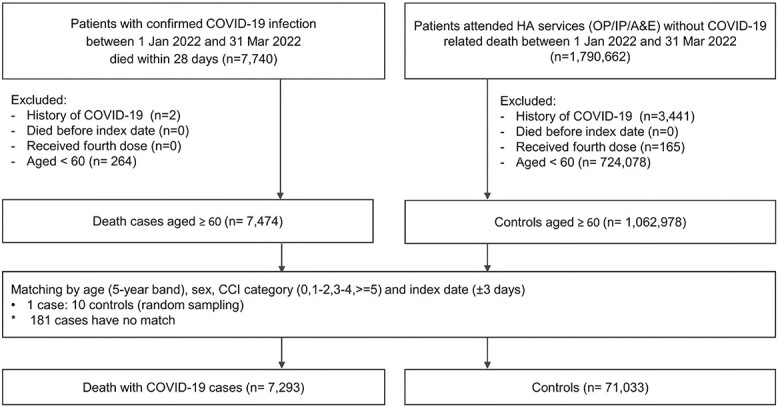
Selection of cases and controls of COVID-19 infection. ^*^PCR = polymerase chain reaction; RAT = rapid antigen test; HA = hospital authority

Figure 1 and Supplementary Figures 1–3, available as Supplementary data at *JTM* online, illustrate the selection of matched cases and controls for the outcome of COVID-19 infection and each COVID-19-related outcome respectively. Among all individuals eligible for study inclusion, 204 901 COVID-19 cases were matched with 819 315 controls; 25 362 hospitalized cases were matched with 252 577 controls; 1228 cases of severe complications were matched with 12 252 controls and 7458 cases of mortality were matched with 73 770 controls. Table 1 shows characteristics of eligible cases and matched controls.

**Table 1:** Baseline characteristics

	COVID-19 infection		COVID-19-related hospitalization		COVID-19-related severe complications		COVID-19-related mortality	
	Case	Control	Case	Control	Case	Control	Case	Control
*N*	176 780	738 925	24 130	238 060	1184	11 469	7293	71 033
Age, years	72.29 (9.62)	72.00 (9.49)	80.84 (10.52)	80.61 (10.48)	79.54 (9.96)	79.54 (9.96)	85.30 (9.37)	85.02 (9.29)
Sex, male	91 492 (51.8)	382 207 (51.7)	13 239 (54.9)	130 416 (54.8)	724 (61.1)	7001 (61.0)	4295 (58.9)	41 839 (58.9)
Charlson Comorbidity Index	3.49 (1.66)	3.42 (1.59)	4.87 (1.94)	4.77 (1.89)	4.76 (1.98)	4.71 (1.97)	5.58 (2.04)	5.47 (1.95)
Hypertension	87 630 (49.6)	434 924 (58.9)	13 417 (55.6)	161 187 (67.7)	654 (55.2)	7678 (66.9)	4201 (57.6)	50 189 (70.7)
Cancer	9197 (5.2)	31 100 (4.2)	2329 (9.7)	20 703 (8.7)	86 (7.3)	968 (8.4)	710 (9.7)	7500 (10.6)
Diabetes mellitus	56 682 (32.1)	268 480 (36.3)	9137 (37.9)	111 402 (46.8)	481 (40.6)	5341 (46.6)	2786 (38.2)	34 433 (48.5)
Chronic kidney disease	6486 (3.7)	28 293 (3.8)	2548 (10.6)	23 901 (10.0)	151 (12.8)	1110 (9.7)	1054 (14.5)	10 456 (14.7)
Respiratory disease	8011 (4.5)	25 489 (3.4)	2425 (10.0)	16 439 (6.9)	125 (10.6)	786 (6.9)	853 (11.7)	6998 (9.9)
Coronary heart disease	14 789 (8.4)	63 030 (8.5)	3286 (13.6)	27 725 (11.6)	164 (13.9)	1368 (11.9)	1402 (19.2)	9592 (13.5)
Stroke	16 602 (9.4)	61 396 (8.3)	4907 (20.3)	39 753 (16.7)	215 (18.2)	1896 (16.5)	1988 (27.3)	15 657 (22.0)
Heart failure	6250 (3.5)	17 869 (2.4)	2565 (10.6)	15 374 (6.5)	138 (11.7)	748 (6.5)	1157 (15.9)	7019 (9.9)
Dementia	2551 (1.4)	4932 (0.7)	1414 (5.9)	5165 (2.2)	61 (5.2)	290 (2.5)	683 (9.4)	2418 (3.4)
Renin-angiotensin-system agents	54 489 (30.8)	267 355 (36.2)	8949 (37.1)	101 621 (42.7)	458 (38.7)	4854 (42.3)	2605 (35.7)	31 111 (43.8)
Beta blockers	35 315 (20.0)	165 165 (22.4)	6467 (26.8)	61 007 (25.6)	350 (29.6)	2900 (25.3)	2169 (29.7)	18 397 (25.9)
Calcium channel blockers	76 337 (43.2)	385 593 (52.2)	12 002 (49.7)	138 935 (58.4)	578 (48.8)	6544 (57.1)	3838 (52.6)	42 103 (59.3)
Diuretics	16 289 (9.2)	53 128 (7.2)	5367 (22.2)	28 825 (12.1)	312 (26.4)	1394 (12.2)	2513 (34.5)	10 610 (14.9)
Nitrates	11 124 (6.3)	38 424 (5.2)	2858 (11.8)	18 777 (7.9)	149 (12.6)	921 (8.0)	1071 (14.7)	6698 (9.4)
Lipid lowering agents	77 319 (43.7)	393 613 (53.3)	11 724 (48.6)	141 034 (59.2)	557 (47.0)	6794 (59.2)	3306 (45.3)	42 061 (59.2)
Oral anticoagulants	6463 (3.7)	22 355 (3.0)	2002 (8.3)	12 972 (5.4)	102 (8.6)	588 (5.1)	700 (9.6)	4824 (6.8)
Antiplatelets	36 744 (20.8)	154 366 (20.9)	8795 (36.4)	71 811 (30.2)	431 (36.4)	3477 (30.3)	3270 (44.8)	24 755 (34.8)
Immunosuppressants	1300 (0.7)	4881 (0.7)	413 (1.7)	1142 (0.5)	36 (3.0)	61 (0.5)	474 (6.5)	259 (0.4)
Anti-diabetic drugs	42 653 (24.1)	200 707 (27.2)	6546 (27.1)	80 727 (33.9)	348 (29.4)	3894 (34.0)	1841 (25.2)	23 971 (33.7)
Insulin	8445 (4.8)	27 354 (3.7)	2548 (10.6)	12 560 (5.3)	140 (11.8)	645 (5.6)	2031 (27.8)	4201 (5.9)
Unvaccinated	41 126 (23)	141 732 (19)	12 814 (53)	72 406 (30)	627 (53)	3476 (30)	5017 (69)	24 920 (35)
1 dose only								
BNT162b2	5650 (3)	27 681 (4)	679 (3)	7667 (3)	45 (4)	412 (4)	121 (2)	2055 (3)
CoronaVac	28 704 (16)	94 500 (13)	5277 (22)	40 150 (17)	263 (22)	1953 (17)	1411 (19)	13 675 (19)
2 doses only					.			
All BNT162b2	18 179 (15)	94 655 (13)	938 (4)	22 636 (10)	38 (3)	991 (9)	80 (1)	5316 (7)
All CoronaVac	41 896 (24)	157 401 (21)	3354 (14)	52 571 (22)	166 (14)	2430 (21)	605 (8)	15 529 (22)
3 doses								
All BNT162b2	11 497 (7)	91 154 (12)	275 (1)	15 624 (7)	17 (1)	787 (7)	16 (0)	3404 (5)
All CoronaVac	23 786 (13)	98 300 (13)	646 (3)	21 038 (9)	25 (2)	1098 (10)	36 (0)	4947 (7)
B-B-C	164 (0)	615 (0)	4 (0)	178 (0)	0 (0)	11 (0)	0 (0)	28 (0)
C-C-B	5778 (3)	32 887 (4)	143 (1)	5790 (2)	3 (0)	311 (3)	7 (0)	1159 (2)
Time since the last dose, days	33 (88)	29 (77)	25 (42)	26 (57)	24 (37)	25 (59)	27 (28)	24 (40)

All parameters except time since the last dose are expressed in frequency (percentage), mean (SD) or median (IQR).

**Figure 2:**
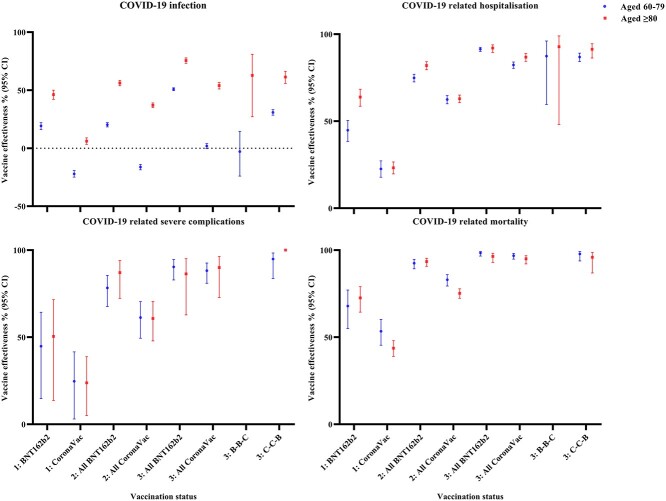
Vaccine effectiveness against COVID-19-related outcomes and mortality among individuals with different vaccination status.

The number of cases and controls, vaccine effectiveness (VE (95 CI%)%) for each outcome was summarized in Table 2 and Supplementary Table 1, available as Supplementary data at *JTM* online, and illustrated in Figure 2. In general, both BNT162b2 and CoronaVac vaccination were associated with a significantly higher VE against COVID-19 infection and related outcomes compared to unvaccinated individuals, except for the younger (age 60–79) CoronaVac recipients. In the age group of 60–79, VE against COVID-19 infection increased substantially from 19.2% (16.2–2.0%) and −22.1% (−24.9–19.3%) after one dose of BNT162b2 or CoronaVac, respectively, to 50.9% (49.6–52.1%) and 1.9% (−0.3–4.0%) after three doses of BNT162b2 or CoronaVac, respectively. A similar dose–response relationship was observed, with more doses of vaccine administered leading to a higher VE against hospital admission, ICU admission or use of ventilatory support, and all-cause mortality after COVID-19 infection. VE against hospital admission, ICU admission or use of ventilatory support, and all-cause mortality after COVID-19 infection in the younger age group were 91.4% (90.1–92.5%), 90.4% (82.9–94.6%) and 98.4% (96.6–99.2%) after three doses of BNT162b2; and were 82.3% (80.4–84.0%), 88.2% (81.0–92.6%) and 96.8% (94.8–98.1%) after three doses of CoronaVac, respectively.

**Table 2:** Vaccine effectiveness against COVID-19-related outcomes and mortality among individuals with different vaccination status

	Unvaccinated	1 dose only		2 doses only		3 doses			
		BNT162b2	CoronaVac	All BNT162b2	All CoronaVac	All BNT162b2	All CoronaVac	B-B-C	C-C-B
COVID-19 infection									
Age 60–79	REF	19.2 (16.2–22.0)	−22.1 (−24.9–19.3)	20.3 (18.4–22.1)	−16.2 (−18.6–13.9)	50.9 (49.6–52.1)	1.9 (−0.3–4.0)	−2.9 (−24.0–14.6)	30.9 (28.5–33.2)
Age ≥ 80	REF	46.2 (42.1–50.0)	6.1 (3.3–8.9)	56.2 (53.8–58.5)	37.1 (35.1–39.1)	75.6 (73.2–77.8)	54.0 (51.1–56.6)	62.8 (27.1–81.0)	61.4 (55.8–66.3)
COVID-19-related hospitalization									
Age 60–79	REF	44.8 (38.4–50.5)	22.6 (17.8–27.2)	74.9 (72.6–77.0)	62.5 (60.0–64.7)	91.4 (90.1–92.5)	82.3 (80.4–84.0)	87.4 (59.6–96.1)	86.9 (84.3–89.1)
Age ≥ 80	REF	63.9 (58.6–68.4)	23.2 (19.7–26.6)	82.0 (79.6–84.2)	62.9 (60.8–65.0)	92.0 (89.5–93.9)	86.8 (84.4–88.9)	92.8 (48.1–99.0)	91.3 (86.3–94.5)
COVID-19-related severe complications									
Age 60–79	REF	44.8 (14.8–64.3)	24.7 (3.0–41.6)	78.3 (67.6–85.5)	61.3 (49.4–70.4)	90.4 (82.9–94.6)	88.2 (81.0–92.6)	NA	94.9 (83.7–98.4)
Age ≥ 80	REF	50.5 (13.7–71.6)	23.8 (5.0–38.8)	87.1 (72.3–94.0)	60.8 (47.9–70.5)	86.4 (62.8–95.1)	90.0 (72.8–96.3)	NA	NA
COVID-19-related mortality									
Age 60–79	REF	67.9 (54.9–77.1)	53.4 (45.4–60.2)	92.5 (89.3–94.7)	83.0 (79.4–85.9)	98.4 (96.6–99.2)	96.8 (94.8–98.1)	NA	97.8 (93.8–99.2)
Age ≥ 80	REF	72.6 (64.4–79.0)	43.7 (39.0–48.0)	93.4 (90.7–95.3)	75.2 (72.3–77.8)	96.4 (92.9–98.2)	95.0 (92.1–96.8)	NA	95.9 (86.9–98.7)

All parameters are expressed in vaccination effectiveness (95% confidence interval).

The dose–response relationship observed in the age group of 80 years or above was in line with the younger age group, showing higher effectiveness against infection, in which VE against COVID-19 infection after only one dose of BNT162b2 and CoronaVac were 46.2% (42.1–50.0%) and 6.1% (3.3–8.9%), respectively, and increased significantly to 56.2% (53.8–58.5%) and 37.1% (35.1–39.1%) after two doses of BNT162b2 and CoronaVac, then 75.6% (73.2–77.8%), 54.0% (51.1–56.6%) after three doses of BNT162b2 and CoronaVac vaccinations, respectively. A consistent pattern was observed for other outcomes in that age group, with more doses of vaccine administered leading to a higher VE against hospital admission, ICU admission or the need of ventilatory support, and all-cause mortality after COVID-19 infection. VE against hospital admission, ICU admission or use of ventilatory support, and all-cause mortality after COVID-19 infection in the older age group were 92.0% (89.5–93.9%), 86.4% (62.8–95.1%) and 96.4% (92.9–98.2%) after three doses of BNT162b2; and were 86.8% (84.4–88.9%), 90.0% (72.8–96.3%) and 95.0% (92.1–96.8%) after three doses of CoronaVac, respectively.

However, due to a limited number of patients who received a CoronaVac booster after two doses of BNT162b2, VE against severe complications or mortality in this group of people could not be estimated. Similarly, VE against severe complications in people aged 80 or above who received a BNT162b2 booster after two doses of CoronaVac could not be estimated because of the same reason. In the present study, a primary series of CoronaVac with BNT162b2 booster seems to have higher VE against infection than homologous CoronaVac [Aged 60–79: 30.9% (28.5–33.2%), aged 80 or older: 61.4% (55.8–66.3%)], but no significant difference in their VE against COVID-19-related hospitalization, severe complications and mortality was detected.

The dose–response relationship observed in the results of the sensitivity analyses echoes that derived from the main analysis (Supplementary Tables 2–4 available as Supplementary data at *JTM* online), indicating that the increase in VE with the number of vaccine doses was unlikely to be affected by our selection criteria, regardless of the duration since the last dose of vaccination or the definition of a confirmed COVID-19 case. In the subgroup analyses as shown in Table 3, a similar dose–response relationship was established after stratification by sex and Charlson Comorbidity Index. VE against COVID-19 infection increased substantially after the third dose in the younger age group (60–79 years), whereas VE increased with each additional dose of vaccination in the older age group (80 years or above). VE against other COVID-19-related outcomes increased with the number of vaccine doses administered in both age groups.

**Table 3:** Vaccine effectiveness against COVID-19-related outcomes and mortality among individuals with different vaccination status stratified by age and gender

	1 dose only		2 doses only		3 doses			
	BNT162b2	CoronaVac	All BNT162b2	All CoronaVac	All BNT162b2	All CoronaVac	B-B-C	C-C-B
COVID infection								
Age 60–79								
Male	19.5 (15.2–23.6)	−24.5 (−28.5–20.5)	17.1 (14.3–19.7)	−16.9 (−20.3–13.6)	48.3 (46.5–50.1)	2.5 (−0.5–5.4)	−20.0 (−49.2–3.4)	28.9 (25.8–31.9)
Female	19.3 (16.2–22.2)	−20.0 (−23.9–16.2)	23.4 (20.8–25.9)	−15.9 (−19.2–12.7)	53.9 (52.0–55.7)	0.4 (−2.9–3.6)	29.2 (−2.1–50.9)	33.3 (29.6–36.8)
CCI < 4	−3.5 (−25.6–14.8)	−23.6 (−26.6–20.8)	19.0 (17.1–21.0)	−18.9 (−21.4–16.5)	50.0 (48.6–51.3)	0.3 (−2.0–2.5)	−3.6 (−24.9–14.1)	29.6 (27.2–32.0)
CCI ≥ 4	0	−5.4 (−19.5–7.0)	38.7 (28.7–47.4)	33.6 (24.6–41.5)	77.6 (70.9–82.7)	47.7 (34.3–58.4)	NA (NA–NA)	68.4 (52.8–78.9)
Age ≥ 80								
Male	47.5 (42.0–52.5)	10.8 (6.8–14.6)	56.2 (52.9–59.2)	40.3 (37.5–42.9)	72.2 (68.9–75.2)	51.8 (48.0–55.4)	78.5 (39.6–92.3)	58.5 (51.1–64.8)
Female	45.0 (38.7–50.8)	1.9 (−2.2–5.9)	56.5 (52.7–60.0)	34.0 (31.1–36.9)	81.2 (77.7–84.1)	57.4 (53.1–61.3)	30.6 (−72.2–72.1)	66.8 (57.8–73.9)
CCI < 4	45.6 (41.3–49.5)	4.7 (1.7–7.6)	55.6 (53.0–58.0)	35.6 (33.4–37.6)	75.5 (73.1–77.7)	53.5 (50.6–56.2)	66.3 (31.9–83.3)	60.7 (54.9–65.8)
CCI ≥ 4	49.0 (25.5–65.2)	20.8 (8.1–31.8)	70.1 (59.7–77.8)	63.5 (56.3–69.6)	74.0 (53.4–85.5)	61.7 (42.0–74.7)	NA	84.2 (58.6–94.0)
COVID-19-related hospitalization								
Age 60–79								
Male	42.9 (34.4–50.2)	17.2 (10.5–23.4)	75.7 (72.8–78.3)	62.9 (59.8–65.8)	90.7 (88.9–92.1)	82.2 (79.9–84.2)	89.8 (57.7–97.5)	86.0 (82.6–88.7)
Female	47.1 (36.5–55.9)	29.6 (22.4–36.2)	73.4 (69.5–76.8)	61.4 (57.5–65.0)	92.6 (90.4–94.3)	82.1 (78.8–85.0)	76.2 (−79.5–96.9)	88.8 (83.8–92.3)
CCI < 4	49.1 (42.5–54.9)	21.7 (16.4–26.7)	76.6 (74.3–78.7)	62.0 (59.4–64.4)	92.0 (90.7–93.1)	82.7 (80.8–84.4)	87.8 (60.7–96.2)	87.4 (84.7–89.6)
CCI ≥ 4	22.6 (−4.5–42.7)	25.4 (10.3–38.0)	55.9 (43.4–65.6)	70.5 (62.9–76.5)	85.4 (76.9–90.8)	76.4 (63.6–84.7)	NA (NA–NA)	77.9 (55.5–89.0)
Age ≥ 80								
Male	63.2 (56.0–69.2)	25.1 (20.1–29.8)	82.5 (79.4–85.2)	66.4 (63.5–69.0)	90.2 (86.6–92.8)	85.7 (82.4–88.4)	90.3 (29.6–98.7)	90.5 (83.8–94.4)
Female	64.9 (56.7–71.5)	21.0 (15.8–25.8)	81.3 (77.2–84.6)	58.9 (55.5–62.1)	95.1 (91.3–97.2)	88.7 (84.7–91.6)	NA	93.0 (83.1–97.1)
CCI < 4	63.2 (57.6–68.0)	22.1 (18.4–25.7)	82.0 (79.4–84.2)	62.1 (59.8–64.3)	92.5 (90.0–94.4)	87.3 (84.8–89.4)	92.8 (47.9–99.0)	91.8 (86.8–94.9)
CCI ≥ 4	71.1 (49.5–83.5)	31.0 (17.3–42.3)	86.0 (76.1–91.7)	74.8 (67.2–80.6)	79.3 (50.9–91.3)	74.4 (53.0–86.0)	NA	75.9 (−1.6–94.3)
COVID-19-related severe complications								
Age 60–79								
Male	42.3 (2.5–65.9)	25.6 (−1.8–45.6)	85.3 (74.1–91.6)	58.7 (42.9–70.1)	88.1 (77.9–93.6)	88.7 (80.1–93.6)	NA	93.6 (79.2–98.0)
Female	47.7 (−18.5–76.9)	22.6 (−21.6–50.7)	58.0 (22.9–77.1)	63.5 (40.2–77.7)	97.0 (78.1–99.6)	86.3 (66.6–94.4)	NA	NA
CCI < 4	49.4 (19.1–68.4)	23.5 (−0.4–41.7)	80.9 (70.6–87.6)	61.7 (49.2–71.1)	91.0 (83.4–95.1)	89.4 (82.5–93.6)	NA	96.8 (86.8–99.2)
CCI ≥ 4	−10.9 (−306.0–69.7)	77.4 (29.8–92.7)	54.7 (−137.0–91.4)	70.6 (9.1–90.5)	89.3 (−3.6–98.9)	50.0 (−212.4–92.0)	NA	75.2 (−366.1–98.7)
Age ≥ 80								
Male	61.5 (17.4–82.0)	25.9 (−0.2–45.2)	85.8 (64.6–94.3)	64.9 (47.9–76.3)	84.5 (50.0–95.2)	93.0 (71.4–98.3)	NA	NA
Female	31.7 (−53.3–69.6)	21.6 (−8.5–43.3)	89.0 (54.6–97.3)	55.0 (31.9–70.3)	91.2 (30.4–98.9)	82.5 (27.3–95.8)	NA	NA
CCI < 4	50.4 (9.6–72.8)	20.9 (0.0–37.4)	85.0 (67.8–93.0)	60.4 (46.6–70.7)	85.6 (60.3–94.8)	89.0 (70.0–96.0)	NA	NA
CCI ≥ 4	17.7 (−350.1–84.9)	54.7 (0.3–79.4)	NA	57.5 (−20.8–85.0)	NA	NA	NA	NA
COVID-19-related mortality								
Age 60–79								
Male	63.6 (46.6–75.2)	51.1 (41.1–59.5)	92.8 (89.0–95.3)	83.4 (79.1–86.8)	98.5 (96.3–99.4)	96.2 (93.6–97.8)	NA	98.1 (93.6–99.4)
Female	78.2 (53.7–89.7)	58.4 (43.4–69.4)	91.6 (84.5–95.4)	82.5 (75.1–87.7)	98.0 (91.7–99.5)	98.4 (93.6–99.6)	NA	96.3 (72.9–99.5)
CCI < 4	71.4 (56.9–81.0)	50.5 (40.4–58.9)	94.9 (92.1–96.7)	83.1 (78.9–86.4)	98.5 (96.5–99.3)	97.8 (96.0–98.8)	NA	97.8 (93.5–99.2)
CCI ≥ 4	58.5 (18.8–78.8)	63.3 (46.0–75.0)	74.1 (53.8–85.5)	83.8 (73.9–89.9)	98.1 (85.7–99.7)	85.8 (64.0–94.4)	NA	NA
Age ≥ 80								
Male	74.0 (63.9–81.3)	47.5 (41.4–53.0)	94.6 (91.6–96.5)	76.4 (72.7–79.6)	95.8 (91.1–98.0)	94.6 (90.8–96.8)	NA	96.0 (83.6–99.0)
Female	70.0 (53.2–80.8)	39.3 (31.9–46.0)	90.9 (84.7–94.6)	73.8 (69.1–77.8)	98.4 (88.7–99.8)	96.3 (89.9–98.6)	NA	96.1 (67.9–99.5)
CCI < 4	73.4 (64.3–80.2)	41.9 (36.7–46.7)	93.6 (90.7–95.6)	74.3 (71.1–77.1)	96.4 (92.7–98.2)	95.1 (91.9–97.0)	NA	97.3 (88.4–99.4)
CCI ≥ 4	70.6 (41.3–85.3)	64.4 (52.8–73.1)	93.7 (84.3–97.4)	86.7 (78.8–91.6)	NA	98.0 (84.3–99.7)	NA	66.6 (−152.5–95.6)

All parameters are expressed in vaccination effectiveness (95% confidence interval).

## Discussion

This study is one of the first epidemiological studies that focus on the older population, investigating the effectiveness of BNT162b2 and CoronaVac against COVID-19 infection and related outcomes in a predominantly Omicron BA.2 outbreak in Hong Kong. Our results show an increasing VE against COVID-19 infection with each additional dose of vaccine, except in the younger group (60–79 years) who received CoronaVac. Greater protection towards severe COVID-19 outcomes and mortality was demonstrated. Our findings provide evidence to the dose–response relationship between the vaccine effectiveness and the number of doses received, revealing improved effectiveness with the third dose of vaccination in the older population, and thus encouraging the elderly to get vaccinated and boosted to enhance the overall preparedness for COVID-19.

Our finding of the highest vaccine effectiveness against Omicron BA.2 infection after three doses of BNT162b2 in older adults is in line with prior studies which support the effectiveness of booster shots against Omicron in the general population. A UK study conducted using population-based data reported waning immunity of vaccination after receiving only two doses of BNT162b2 and the effectiveness was picked up to a new high by a third booster shot, demonstrating the effectiveness of three doses of BNT162b2 against symptomatic disease caused by Omicron BA.2.^56^ Similar result was also reported by a randomized controlled trial, in which the relative efficacy against COVID-19 infection (variant unspecified) provided by three doses of BNT162b2 vaccine was 95.3% compared to those who only received two doses.^57^ Conversely, the effectiveness of CoronaVac has been studied to an even lesser extent. Descriptive observational studies involved populations immunized with only two doses of the CoronaVac,^18^^,^^19^ leaving the effectiveness of three doses of CoronaVac remains to be addressed. Bridging this knowledge gap, our study, which determined the effectiveness after the first, second, and third dose of CoronaVac among older adults, demonstrated a positive relationship between the number of doses and the related vaccine effectiveness. The strongest protection was observed after three doses of CoronaVac and this finding is compatible with a study, which reported a significant elevation in the neutralizing antibody response against Omicron in the sera from people receiving the third dose of an inactivated vaccine,^58^ hence supporting the importance of booster dose administration in this patient group.

Apart from illustrating the effectiveness of three doses of vaccination, this study also allowed for a comparison between mRNA and inactivated vaccines against the Omicron BA.2 infection in the older population. The present study showed superior effectiveness of three doses of BNT162b2 compared to three doses of CoronaVac against any COVID-19 infection. This is consistent with findings from a serological study conducted in Turkey, which revealed higher neutralizing antibody levels three months after the boosting of BNT162b2 than that of CoronaVac.^59^ In terms of real-world evidence, a simulation study in Hong Kong also showed higher effectiveness among adults aged 60 years or older with BNT162b2 compared with CoronaVac.^23^ Additionally, an observational study conducted in Singapore even concluded a 2.4-fold infection risk among CoronaVac recipients compared to BNT162b2 recipients.^60^ Our study was consistent with the previous studies which suggested stronger protection from BNT162b2 against COVID-19 infection, and further extended the current evidence beyond the general population to the older population. Meanwhile, their difference in effectiveness against COVID-19-related severe complications and mortality is less evident, and this concurs with earlier studies that compared inactivated and mRNA vaccines.^61^ Besides, the superior effectiveness of BNT162b2 was also observed in a heterologous booster dose of BNT162b2 after two doses of CoronaVac in the older population, when compared with the effectiveness of a homologous booster dose of CoronaVac. This aligns with a prior study which revealed a higher rise in antibody concentrations in BNT162b2 booster recipients as opposed to homologous booster recipients after two doses of CoronaVac in Brazil,^62^^,^^63^ and similar findings against the Omicron variant from a study in Hong Kong.^64^ Given that limited people received heterologous boosters in this study, further studies are warranted to confirm our findings. By and large, both homologous and heterologous boosters were effective in protecting against severe COVID-19 diseases. In fact, our findings show the effectiveness of both vaccines against COVID-19 infection and COVID-19-related outcomes during the Omicron BA.2-dominant outbreak among the older population.

On another note, the increased transmissibility of the Omicron variant might decrease the effectiveness of vaccines,^65^ whereas transmission is also influenced by human behaviour, particularly through prevention strategies. Besides, the vaccine uptake among the elderly in Hong Kong during the inclusion period was suboptimal, which could have resulted in a high mortality rate recorded locally^66^ despite the high VE against COVID-19 mortality demonstrated in our study. In comparing the VE among individuals aged between 60 and 79 and those aged 80 or above, we observed higher VE against COVID-19 infection and COVID-19-related hospitalization in the older age group. In addition to that, our results yielded negative VEs against COVID-19 infection in vaccinated individuals aged 60–79 with one to two doses of CoronaVac. This could be possibly explained by their different behaviours, as younger elderly may have more social activities and thus have a higher risk of contracting an infection.^67^ Furthermore, unvaccinated persons were prohibited from entering specified premises, including but not limited to restaurants and bars, due to the enforcement of the Vaccine Pass in Hong Kong.^68^ As a result, this group of people might have less frequent social interactions and hence be prevented from getting an infection, seemingly diluting the effect of vaccination in reducing COVID-19 infections. In our study, negative VEs were demonstrated in CoronaVac recipients only, and this is consistent with a previous serological study that reported low IgG antibody levels in people who have received one to two doses of CoronaVac.^69^ It should be noted that the protection against severe COVID-19 disease offered by the vaccines might be affected by some other factors which remain unexplored, and further studies are needed to confirm our findings on the potential impact of age on vaccine immunogenicity. On the other hand, we displayed a consistently stable VE against severe COVID-19 outcomes including hospitalization and mortality in both age groups. With the emergence of new variants of increased transmissibility and reduced sensitivity to vaccine-elicited antibodies, members of the public, especially vulnerable groups whose magnitude and quality of immune responses may be suboptimal, are highly encouraged to get vaccinated.

The main strength of this study is being one of the first to evaluate the real-world effectiveness of an mRNA (BNT162b2) and inactivated virus (CoronaVac) vaccine against the Omicron BA.2 variant with regards to clinical outcomes in the older population aged 60 and above. Our results support the need for a booster vaccination in maintaining substantial protection against the Omicron variant while providing additional information on vaccine effectiveness when choosing the type of vaccine to receive for the booster dose in older people. Still, our study had a few limitations. First, the present study did not document asymptomatic COVID-19 infection. This may have resulted in patients with prior asymptomatic COVID-19 infection being included in the study as a new COVID-19 case. For the same reason, misclassification of asymptomatic infection as controls might have introduced bias in estimating the effectiveness of vaccines against COVID-19 infection. Asymptomatic cases can be better captured in nationwide screening, which is not conducted in most places worldwide including Hong Kong, where we acquired the data for analysis. For the outcomes of hospitalization and death, it is less likely that asymptomatic infection would have biased the results as most hospitalized patients underwent PCR testing on admission. Second, there was a clear preference for BNT162b2 boosting which led to a limited number of individuals receiving CoronaVac booster following the primary series of COVID-19 vaccines. Hence, the risk of infection-related complications and mortality in this group of people could not be estimated. Third, the COVID-19-related severe complications were defined merely based on the procedure codes requiring medical staff to manually enter into electronic medical records, plus the ICU beds were over-utilized amid the fifth Covid-19 wave dominant by the Omicron variant, suggesting the potential for underdiagnosis for this outcome. Fourth, waning immunity after vaccination may have affected our results. Further studies should be conducted to evaluate the duration of protection provided by COVID-19 vaccination. Moreover, this study did not examine the effect of hybrid immunity because patients with prior COVID-19 infection were excluded. Further research in this regard is needed as prior studies pointed out a stronger antibody response after vaccination in previously infected people.^70^^,^^71^ Lastly, other unmeasured factors, such as different health-seeking behaviours, might have confounded the relationship between vaccine status or vaccine type and the risk of contracting COVID-19 infection.

## Conclusion

Our findings revealed a clear positive dose–response relationship between the number of COVID-19 vaccine doses (BNT162b2 or CoronaVac) and the magnitude of protection in terms COVID-19-related hospitalization, severe complications, and mortality, among those aged 60 and above during the Omicron BA.2-dominant pandemic. A similar relationship was observed in terms of VE against COVID-19 infection, except for the age group of 60–79 years who received CoronaVac. It is essential for the vulnerable older population to be vaccinated with a booster dose of either vaccine to increase protection against Omicron-associated COVID-19 infection and severe outcomes.

## Supplementary Material

supplementary_figures_taac119

supplementary_tables_taac119

## Data Availability

Data will not be made available to others because the data custodians have not given permission.
