# Supplementary material for: Effectiveness of BNT162b2 and CoronaVac vaccinations against SARS-CoV-2 omicron infection in people aged 60 years or above: a case–control study
Source: J Travel Med. 2022 Oct 17;29(8):taac119. doi: 10.1093/jtm/taac119 (PMC9619717; doi:10.1093/jtm/taac119)

Supplementary figure 1. Selection of cases and controls of COVID-19 related hospitalisation


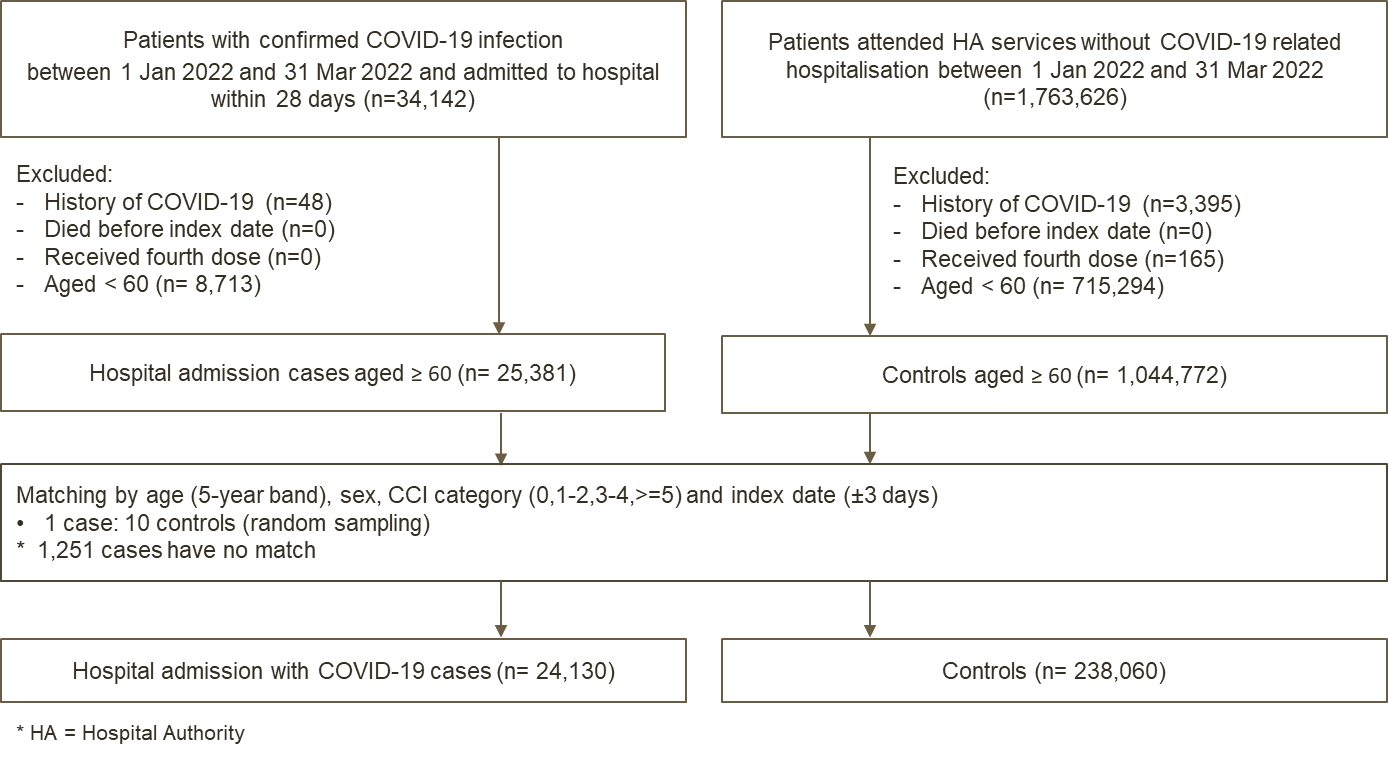


Supplementary figure 2. Selection of cases and controls of COVID-19 related severe complications


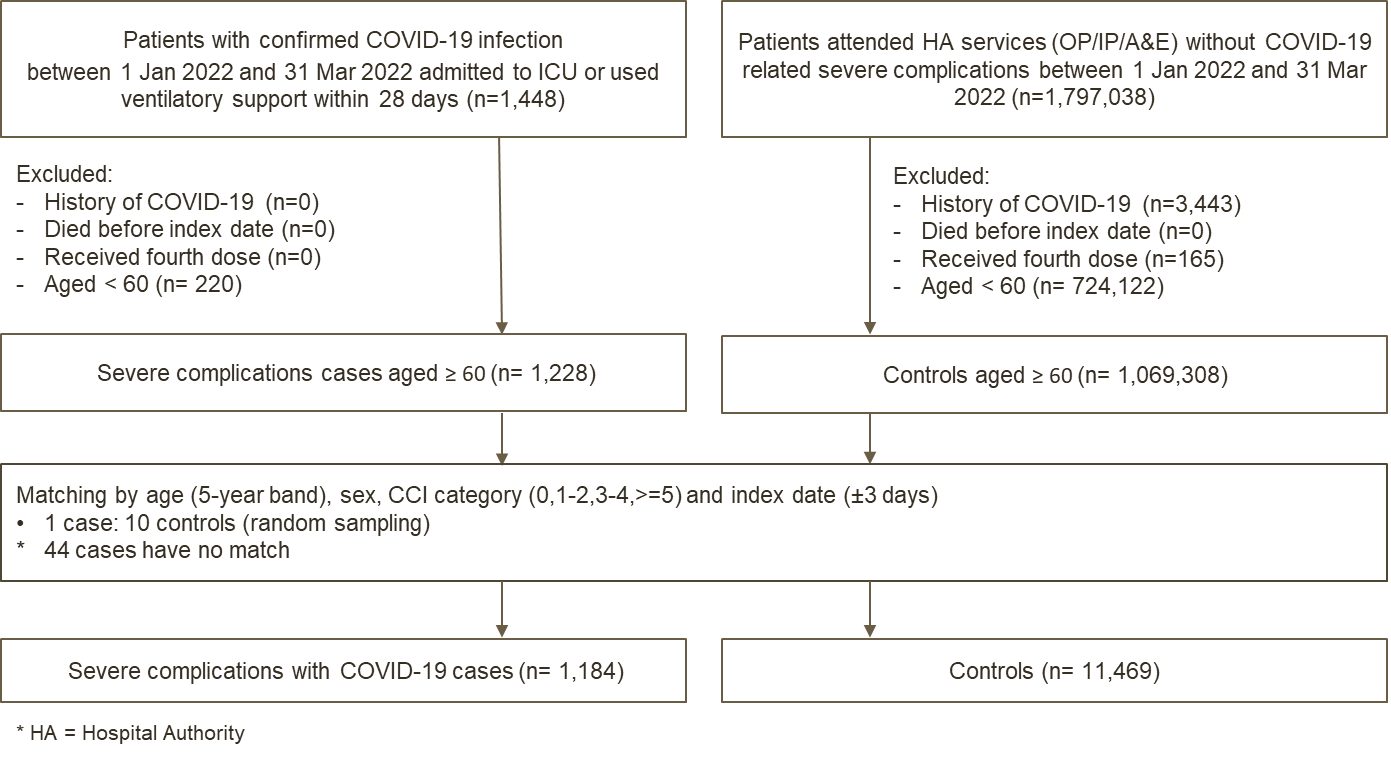


Supplementary figure 3. Selection of cases and controls of COVID-19 related death


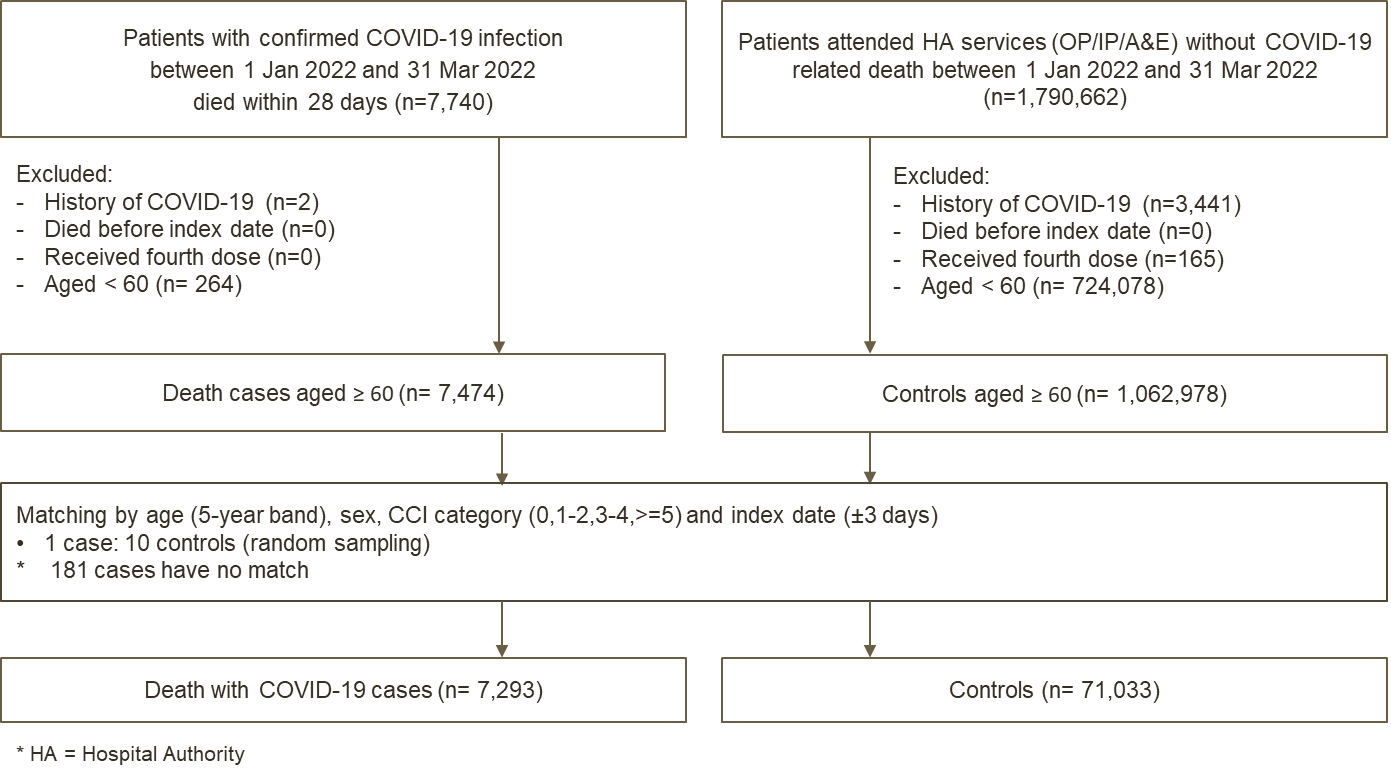

Supplement: supplementary_figures_taac119 [file supplementary_figures_taac119.docx]
