# Supplementary material for: Effectiveness of BNT162b2 and CoronaVac vaccinations against SARS-CoV-2 omicron infection in people aged 60 years or above: a case–control study
Source: J Travel Med. 2022 Oct 17;29(8):taac119. doi: 10.1093/jtm/taac119 (PMC9619717; doi:10.1093/jtm/taac119)
Supplement: supplementary_tables_taac119 [file supplementary_tables_taac119.docx]

Supplementary Table 1. Number of events among individuals with different vaccination status

|  | Unvaccinated | 1 dose only | | 2 doses only | | 3 doses | | | |
| --- | --- | --- | --- | --- | --- | --- | --- | --- | --- |
|  |  | BNT162b2 | CoronaVac | All BNT162b2 | All CoronaVac | All BNT162b2 | All CoronaVac | B-B-C | C-C-B |
| *COVID-19 infection* |  |  |  |  |  |  |  |  |  |
| Age 60-79 |  |  |  |  |  |  |  |  |  |
| Case | 22488 | 4727 | 19692 | 16425 | 34746 | 10982 | 22327 | 154 | 5527 |
| Control | 85121 | 22484 | 64526 | 82061 | 121047 | 84947 | 88634 | 545 | 30944 |
| Age ≥ 80 |  |  |  |  |  |  |  |  |  |
| Case | 18638 | 923 | 9012 | 1754 | 7150 | 515 | 1459 | 10 | 251 |
| Control | 56611 | 5197 | 29974 | 12594 | 36354 | 6207 | 9666 | 70 | 1943 |
| *COVID-19 related hospitalisation* | |  |  |  |  |  |  |  |  |
| Age 60-79 |  |  |  |  |  |  |  |  |  |
| Case | 4530 | 444 | 2102 | 682 | 1741 | 221 | 505 | 3 | 124 |
| Control | 19687 | 3509 | 12890 | 13237 | 23013 | 11447 | 14070 | 92 | 4394 |
| Age ≥ 80 |  |  |  |  |  |  |  |  |  |
| Case | 8284 | 235 | 3175 | 256 | 1613 | 54 | 141 | 1 | 19 |
| Control | 52719 | 4158 | 27260 | 9399 | 29558 | 4177 | 6968 | 86 | 1396 |
| *COVID-19-related severe complications* | |  |  |  |  |  |  |  |  |
| Age 60-79 |  |  |  |  |  |  |  |  |  |
| Case | 262 | 30 | 132 | 31 | 102 | 13 | 21 | 0 | 3 |
| Control | 1076 | 226 | 753 | 640 | 1231 | 603 | 822 | 9 | 252 |
| Age ≥ 80 |  |  |  |  |  |  |  |  |  |
| Case | 365 | 15 | 131 | 7 | 64 | 4 | 4 | 0 | 0 |
| Control | 2400 | 186 | 1200 | 351 | 1199 | 184 | 276 | 2 | 59 |
| *COVID-19 related mortality* | |  |  |  |  |  |  |  |  |
| Age 60-79 |  |  |  |  |  |  |  |  |  |
| Case | 1156 | 53 | 346 | 42 | 171 | 7 | 17 | 0 | 4 |
| Control | 3503 | 550 | 2445 | 1985 | 3984 | 1847 | 2364 | 13 | 694 |
| Age ≥ 80 |  |  |  |  |  |  |  |  |  |
| Case | 3861 | 68 | 1065 | 38 | 434 | 9 | 19 | 0 | 3 |
| Control | 21417 | 1505 | 11230 | 3331 | 11545 | 1557 | 2583 | 15 | 465 |

Supplementary Table 2. Vaccine effectiveness against COVID-19-related outcomes and mortality among individuals after limiting vaccine exposure to at least 14 days from latest dose

|  | Unvaccinated | 1 dose only | | 2 doses only | | 3 doses | | | |
| --- | --- | --- | --- | --- | --- | --- | --- | --- | --- |
|  |  | BNT162b2 | CoronaVac | All BNT162b2 | All CoronaVac | All BNT162b2 | All CoronaVac | B-B-C | C-C-B |
| *COVID-19 infection* | |  |  |  |  |  |  |  |  |
| Age 60-79 | REF | -16.5  (-22.2 - -11.0) | -42.4  (-46.2 - -38.6) | 17.3  (15.2 - 19.3) | -23.1  (-25.8 - -20.5) | -0.1  (-2.6 - 2.3) | -9.3  (-39.0 - 14.0) | 49.4  (47.8 - 50.9) | -0.1  (-2.6 - 2.3) |
| Age ≥ 80 | REF | 16.6  (7.8 - 24.7) | -9.0  (-13.0 - -5.1) | 54.2  (51.4 - 56.9) | 31.2  (28.7 - 33.6) | 73.8  (70.6 - 76.6) | 49.2  (45.5 - 52.6) | 62.7  (10.1 - 84.5) | 55.8  (48.5 - 62.1) |
| *COVID-19 related hospitalisation* | | |  |  |  |  |  |  |  |
| Age 60-79 | REF | 24.2  (13.0 - 34.0) | 13.3  (7.1 - 19.1) | 74.6  (72.1 - 76.8) | 59.4  (56.6 - 62.0) | 90.4  (88.7 - 91.8) | 81.9  (79.7 - 83.8) | 94.0  (55.5 - 99.2) | 85.5  (82.3 - 88.2) |
| Age ≥ 80 | REF | 43.9  (33.5 - 52.7) | 12.4  (7.6 - 16.9) | 81.0  (78.2 - 83.5) | 58.4  (55.8 - 60.9) | 90.5  (87.0 - 93.1) | 87.3  (84.3 - 89.7) | 85.0  (-10.9 - 98.0) | 88.8  (82.0 - 93.0) |
| *COVID-19-related severe complications* | | |  |  |  |  |  |  |  |
| Age 60-79 | REF | 17.1  (-44.7 - 52.4) | 12.0  (-18.0 - 34.3) | 77.5  (65.7 - 85.2) | 57.7  (43.8 - 68.2) | 89.3  (79.1 - 94.5) | 85.2  (75.8 - 91.0) | NA | 92.2  (74.9 - 97.6) |
| Age ≥ 80 | REF | -6.8  (-106.8 - 44.8) | 1.4  (-27.5 - 23.8) | 82.7  (62.7 - 92.0) | 55.0  (38.6 - 67.0) | 78.7  (40.3 - 92.4) | 88.8  (64.4 - 96.5) | NA | NA |
| *COVID-19 related mortality* | |  |  |  |  |  |  |  |  |
| Age 60-79 | REF | 43.8  (17.4 - 61.8) | 45.0  (34.5 - 53.8) | 92.1  (88.7 - 94.5) | 79.6  (75.0 - 83.3) | 97.9  (95.3 - 99.1) | 96.1  (93.5 - 97.7) | NA | 97.3  (92.1 - 99.1) |
| Age ≥ 80 | REF | 44.4  (25.4 - 58.5) | 29.4  (22.8 - 35.4) | 92.5  (89.3 - 94.8) | 69.4  (65.6 - 72.8) | 96.1  (91.2 - 98.3) | 93.7  (89.5 - 96.2) | NA | 94.0  (80.7 - 98.2) |

VE = vaccination effectiveness; CI = confidence interval.

Supplementary Table 3. Vaccine effectiveness against COVID-19-related outcomes and mortality among individuals without limiting vaccine exposure to at most 180 days from latest dose

|  | Unvaccinated | 1 dose only | | 2 doses only | | 3 doses | | | |
| --- | --- | --- | --- | --- | --- | --- | --- | --- | --- |
|  |  | BNT162b2 | CoronaVac | All BNT162b2 | All CoronaVac | All BNT162b2 | All CoronaVac | B-B-C | C-C-B |
| *COVID-19 infection* | |  |  |  |  |  |  |  |  |
| Age 60-79 | REF | 19.0  (16.1 - 21.8) | -22.7  (-25.5 - -20.0) | 10.3  (8.4 - 12.1) | -29.6  (-32.0 - -27.3) | 2.0  (-0.1 - 4.1) | -7.9  (-29.5 - 10.1) | 51.1  (49.9 - 52.4) | 2.0  (-0.1 - 4.1) |
| Age ≥ 80 | REF | 46.8  (42.7 - 50.5) | 6.0  (3.2 - 8.8) | 53.7  (51.4 - 56.0) | 35.3  (33.3 - 37.2) | 75.5  (73.1 - 77.7) | 53.9  (51.0 - 56.5) | 62.5  (26.8 - 80.8) | 61.3  (55.7 - 66.2) |
| *COVID-19 related hospitalisation* | | |  |  |  |  |  |  |  |
| Age 60-79 | REF | 44.8  (38.5 - 50.4) | 21.7  (16.9 - 26.2) | 72.3  (70.2 - 74.3) | 59.4  (57.0 - 61.6) | 91.3  (90.0 - 92.4) | 82.2  (80.4 - 83.9) | 87.1  (58.7 - 96.0) | 86.7  (84.1 - 89.0) |
| Age ≥ 80 | REF | 63.8  (58.6 - 68.4) | 23.3  (19.8 - 26.7) | 79.5  (77.0 - 81.7) | 62.0  (59.8 - 64.0) | 91.9  (89.4 - 93.8) | 86.7  (84.3 - 88.8) | 92.8  (47.9 - 99.0) | 91.3  (86.2 - 94.5) |
| *COVID-19-related severe complications* | | |  |  |  |  |  |  |  |
| Age 60-79 | REF | 44.1  (13.8 - 63.7) | 25.7  (4.5 - 42.2) | 82.1  (74.1 - 87.6) | 61.5  (50.6 - 69.9) | 90.2  (82.5 - 94.5) | 87.8  (80.4 - 92.4) | NA | 94.8  (83.5 - 98.4) |
| Age ≥ 80 | REF | 49.8  (12.5 - 71.2) | 24.0  (5.3 - 38.9) | 84.3  (70.0 - 91.8) | 56.6  (43.3 - 66.8) | 85.8  (61.2 - 94.8) | 89.8  (72.4 - 96.3) | NA | NA |
| *COVID-19 related mortality* | |  |  |  |  |  |  |  |  |
| Age 60-79 | REF | 66.5  (53.1 - 76.1) | 52.9  (44.9 - 59.7) | 89.6  (86.5 - 92.1) | 81.2  (77.7 - 84.2) | 98.4  (96.6 - 99.3) | 96.8  (94.7 - 98.0) | NA | 97.8  (93.8 - 99.2) |
| Age ≥ 80 | REF | 73.0  (64.9 - 79.3) | 43.5  (38.9 - 47.8) | 91.2  (88.4 - 93.3) | 73.9  (71.0 - 76.5) | 96.4  (92.9 - 98.2) | 95.0  (92.1 - 96.8) | NA | 95.8  (86.7 - 98.7) |

VE = vaccination effectiveness; CI = confidence interval.

Supplementary Table 4. Vaccine effectiveness against COVID-19-related outcomes and mortality among individuals after including RAT positive cases

|  | Unvaccinated | 1 dose only | | 2 doses only | | 3 doses | | | |
| --- | --- | --- | --- | --- | --- | --- | --- | --- | --- |
|  |  | BNT162b2 | CoronaVac | All BNT162b2 | All CoronaVac | All BNT162b2 | All CoronaVac | B-B-C | C-C-B |
| *COVID-19 infection* | |  |  |  |  |  |  |  |  |
| Age 60-79 | REF | 4.1  (0.9 - 7.2) | -27.8  (-30.6 - -25.1) | 18.6  (16.8 - 20.4) | -8.2  (-10.3 - -6.1) | 14.0  (12.2 - 15.9) | 3.7  (-15.8 - 19.9) | 52.9  (51.8 - 54.0) | 14.0  (12.2 - 15.9) |
| Age ≥ 80 | REF | 41.0  (37.0 - 44.7) | -6.7  (-9.7 - -3.7) | 48.1  (45.6 - 50.5) | 28.5  (26.4 - 30.5) | 73.4  (71.1 - 75.4) | 51.0  (48.4 - 53.6) | 40.2  (0.7 - 64.0) | 61.3  (56.4 - 65.7) |
| *COVID-19 related hospitalisation* | | |  |  |  |  |  |  |  |
| Age 60-79 | REF | 44.2  (37.8 - 49.9) | 22.7  (17.9 - 27.2) | 74.7  (72.4 - 76.8) | 63.1  (60.7 - 65.3) | 91.1  (89.8 - 92.2) | 82.7  (80.9 - 84.3) | 86.0  (55.0 - 95.7) | 86.8  (84.2 - 89.0) |
| Age ≥ 80 | REF | 63.3  (58.0 - 67.9) | 23.5  (20.0 - 26.9) | 81.9  (79.5 - 84.0) | 62.8  (60.6 - 64.9) | 92.1  (89.7 - 94.0) | 87.2  (84.8 - 89.2) | 90.5  (31.2 - 98.7) | 91.3  (86.4 - 94.5) |
| *COVID-19-related severe complications* | | |  |  |  |  |  |  |  |
| Age 60-79 | REF | 45.9  (16.8 - 64.9) | 24.5  (2.9 - 41.3) | 78.2  (67.4 - 85.4) | 60.3  (48.3 - 69.6) | 89.8  (81.8 - 94.3) | 88.5  (81.5 - 92.8) | NA | 95.2  (84.5 - 98.5) |
| Age ≥ 80 | REF | 49.3  (11.6 - 71.0) | 24.8  (6.3 - 39.7) | 87.1  (72.3 - 94.0) | 60.8  (47.8 - 70.6) | 85.6  (60.5 - 94.8) | 90.5  (74.1 - 96.5) | NA | NA |
| *COVID-19 related mortality* | |  |  |  |  |  |  |  |  |
| Age 60-79 | REF | 70.9  (59.3 - 79.1) | 55.4  (47.7 - 61.9) | 92.5  (89.5 - 94.6) | 84.4  (81.1 - 87.2) | 98.7  (97.2 - 99.4) | 97.0  (95.0 - 98.1) | NA | 97.5  (93.3 - 99.1) |
| Age ≥ 80 | REF | 75.7  (68.6 - 81.2) | 43.0  (38.4 - 47.3) | 92.9  (90.1 - 95.0) | 74.5  (71.6 - 77.1) | 96.7  (93.4 - 98.3) | 95.4  (92.6 - 97.1) | NA | 96.1  (87.4 - 98.8) |

VE = vaccination effectiveness; CI = confidence interval.
